# 5-Methylcytosine (m^5^C) modification in peripheral blood immune cells is a novel non-invasive biomarker for colorectal cancer diagnosis

**DOI:** 10.3389/fimmu.2022.967921

**Published:** 2022-09-21

**Authors:** Haofan Yin, Zhijian Huang, Shiqiong Niu, Liang Ming, Hongbo Jiang, Liang Gu, Weibin Huang, Jinye Xie, Yulong He, Changhua Zhang

**Affiliations:** ^1^ Digestive Diseases Center, The Seventh Affiliated Hospital of Sun Yat-Sen University, Shenzhen, Guangdong, China; ^2^ Guangdong Provincial Key Laboratory of Digestive Cancer Research, The Seventh Affiliated Hospital of Sun Yat-Sen University, Shenzhen, Guangdong, China; ^3^ Department of Clinical Laboratory, The Seventh Affiliated Hospital of Sun Yat-Sen University, Shenzhen, Guangdong, China; ^4^ Department of Clinical Laboratory, Zhongshan City People's Hospital, The Affiliated Zhongshan Hospital of Sun Yat-Sen University, Zhongshan, China

**Keywords:** 5-Methylcytosine, colorectal cancer, peripheral blood, biomarker, diagnosis

## Abstract

Current non-invasive tumor biomarkers failed to accurately identify patients with colorectal cancer (CRC), delaying CRC diagnosis and thus leading to poor prognosis. Dysregulation of 5-Methylcytosine (m^5^C) RNA has gradually been reported in various cancers, but their role in tumor diagnosis is rarely mentioned. Our study aimed to determine the role of m^5^C methylation modification in blood immune cells for the diagnosis of CRC. Peripheral blood samples were obtained from a total of 83 healthy controls and 196 CRC patients. We observed that m^5^C RNA contents in blood immune cells of CRC patients were markedly enhanced in both training set and validation set. Moreover, levels of m^5^C increased with CRC progression and metastasis but reduced after treatment. Compared with common blood tumor biomarkers, m^5^C levels in peripheral blood immune cells had superior discrimination and reclassification performance in diagnosing CRC. Besides, bioinformatics and qRT-PCR analysis identified increased expression of m^5^C-modified regulators NSUN5 and YBX1 in CRC patients’ blood. A series of animal models and cell co-culture models further demonstrated that CRC tumor cells could increase immune cells’ m^5^C levels and m^5^C-modified regulators. Monocyte was the predominant m^5^C-modified immune cell type in CRC patients’ blood by Gene set variation analysis (GSVA). Taken together, m^5^C methylation modification in peripheral blood immune cells was a promising biomarker for non-invasive diagnosis of CRC.

## Introduction

CRC is one of the most prevalent malignancies of the digestive system and ranks third in incidence and mortality among malignant tumors (1). The incidence of CRC patients in China has been increasing at an annual rate of 4%-8% over the past 30 years, with a trend toward younger patients (2). Early symptoms of CRC are insidious, and more than 50% of CRC patients have already been in the progressive stage when initially diagnosed (3). Colonoscopy is the gold standard for CRC diagnosis, but the uneven distribution of medical resources in China prevents it from becoming a large-scale screening method (4). Moreover, colonoscopy is also rejected by many populations due to its invasiveness and high cost (3). Meanwhile, the commonly applied CRC blood tumor biomarkers, such as CEA, CA19-9, and CA125, are inadequate for the diagnosis of CRC on account of their poor sensitivity, especially in patients with early-stage CRC (5–7). Therefore, finding other simple and effective biomarkers is imperatively needed to improve the diagnosis of CRC patients.

Researchers have discovered the advantages of methylation testing over gene mutation testing as a cancer screening method (8). Widespread differences in methylation patterns exist between normal and tumor cells. Aberrant methylation sites are commonly observed in enhancer and promoter regions of tumor cells, leading to decreased expression of tumor suppressors and increased expression of oncogenes (9, 10). The abnormal methylation status in the tumor immune microenvironment, which participates in the occurrence and development of tumors, has gradually attracted the attention of researchers (11, 12). Three main types of mRNA methylation have been identified as N6-Methyladenosine (m^6^A), 5-Methylcytosine (m^5^C), and N1-Methyladenosine (m^1^A). Current studies focus on m^6^A modification, while little research has been done on m^5^C modification and m^1^A modification due to the difficulty in detecting (13). Our previous article reported that m^6^A contents in blood served as a diagnostic biomarker and therapeutic target for CRC (14). In this study, we would like to further explore whether m^5^C modification can also be utilized as a biomarker for CRC diagnosis.

Similar to m^6^A modification, m^5^C modification is also encoded by a methyltransferase complex comprised of “writers,” “erasers,” and “readers” (15). However, the specific molecules involved are entirely different. The writers of m^5^C mainly consist of NSUN family proteins, and the erasers mainly include TET family proteins (16, 17). The currently reported readers of m^5^C are Aly/REF nuclear export factor (ALYREF) and Y-box binding protein 1 (YBX1) (16). m^5^C modification of RNA is a reversible epigenetic modification that affects the fate of modified RNA molecule by performing critical functions in a variety of biological processes (18). Aberrant activation of super-enhancers and promoters of lncRNAs can be directly or indirectly affected by m^5^C modification in CRC (19). Besides, it is demonstrated that three m^5^C regulators, NSUN6, ALKBH1, and TRDMT1, govern prognosis of CRC patients, acting in synergy with the MAPK signaling pathway (20). The present studies briefly discover the cancer-promoting effect of m^5^C modification in CRC tumor cells, but the role of m^5^C modification in the immune microenvironment of CRC deserves further exploration.

This research reveals the impact of m^5^C modifications in the immune microenvironment of CRC from the perspective of disease diagnosis. Detection of m^5^C levels in peripheral blood immune cells of CRC patients to assess whether it could be used as a novel biomarker in both training set and validation set. m^5^C-modified regulators NSUN5 and YBX1 were responsible for elevated m^5^C levels. We also applied bioinformatics approach to reveal monocyte as the predominant m^5^C-modified immune cell type in peripheral blood of CRC patients.

## Materials and methods

### Human samples

The Institutional Review Board of Zhongshan People’s Hospital approved this retrospective study (IRB number: K2020-20). Between March 2020 and December 2021, peripheral blood samples from 134 CRC patients and 53 healthy control (HC) who had no history of basic or chronic diseases were collected from the Zhongshan People’s Hospital using EDTA anti-coagulation tubes as the training set. 92 CRC patients’ peripheral blood samples were collected when initially diagnosed before surgery or radiochemotherapy. Among them, peripheral blood was collected for the first time on admission and for the second time 14 days after surgery in 25 CRC patients. Another 42 CRC patients had already received treatment at the time of sample collection. Besides, between March 2022 and May 2022, peripheral blood samples of 62 CRC patients and 30 HC were collected from the Sun Yat-sen University Cancer Center (IRB number: 2022.475.01) as the validation set. Mix 0.5 mL of whole blood and 1mL of red blood cell lysate (TIANGEN, Beijing, China) with gentle shaking. After standing at room temperature for 20 minutes, centrifuge at 1000 rpm for 10 minutes. Aspirated and discard the supernatant, added 1mL red blood cell lysate and shaked gently again. After centrifuge at 1000 rpm for 10 minutes, the supernatant was aspirated and discarded, and the remaining white cell pellet was defined as peripheral blood immune cells. At this point the red blood cells and hemoglobin had been completely removed, peripheral blood immune cells were isolated. The residue was taken and dissolved with 1ml triol to stabilize RNA, after which the composite samples were stored at -80°C. All CRC patients were diagnosed basis on histopathology by biopsy or endoscopic examination, and informed consent was obtained for all participants. Ethics approval was obtained from the Ethics Committee of the Zhongshan People’s Hospital and Sun Yat-sen University Cancer Center. The clinical and biological characteristics of the patients were described in **Table 1** and **Supplementary Table 1**.

**Table 1:** Correlation between the levels of m^5^C and clinicopathological characteristics in the training set.

Characteristics	No.of patients	Peripheral blood m^5^C levels % (mean ± SD)	*P* value
Age			
≤60 >60	50 42	0.377 ± 0.051 0.390 ± 0.063	0.268
Gender			
Female Male	34 58	0.385 ± 0.057 0.382 ± 0.056	0.787
Clinical stage			
I-II	30	0.335 ± 0.037	<0.001
III-IV	62	0.406 ± 0.050	
T classification			
T1-T2	32	0.378 ± 0.049	0.500
T3-T4	60	0.386 ± 0.061	
N classification			
N0	30	0.356 ± 0.052	0.001
N1-N2	62	0.396 ± 0.055	
M classification			
M0 M1	62 30	0.359 ± 0.047 0.433 ± 0.039	<0.001
Differentiation			
Poor Moderate/Well	13 79	0.386 ± 0.049 0.382 ± 0.058	0.825
Tumor budding			
Bd1-Bd2 Bd3	11 16	0.364 ± 0.038 0.386 ± 0.061	0.301
HER2 expression			
Negative Positive	26 22	0.388 ± 0.041 0.360 ± 0.058	0.062
KRAS genotyping			
Wild-type Mutation-type	8 8	0.404 ± 0.050 0.417 ± 0.056	0.627
BRAF genotyping			
Wild-type Mutation-type	15 3	0.407 ± 0.054 0.396 ± 0.052	0.763
CEA (ng/mL)			
<5	54	0.382 ± 0.058	0.884
≥5	38	0.384 ± 0.056	
CA125 (ng/mL)			
<35	70	0.386 ± 0.057	0.330
≥35	22	0.372 ± 0.057	
CA19-9 (ng/mL)			
<35 ≥35	68 24	0.387 ± 0.059 0.370 ± 0.048	0.219

### Monocyte isolation

Peripheral blood leukocytes were collected by the method described above, and then CD14^+^ monocytes were isolated using the EasySep Human Monocyte Isolation Kit (Stemcell Technologies, Cologne, Germany). After added Isolation Cocktail to sample, mixed and incubated at room temperature for 5 minutes. After added Magnetic Particles to sample, mixed and incubated at room temperature for 5 minutes. Place the tube into the magnet and incubated at room temperature for 5 minutes. Pick up the magnet, and invert the magnet and tube, pouring off the enriched monocyte suspension into a new tube. Isolated CD14^+^ monocytes were now ready for use.

### CRC mouse model

C57BL/6 mice and BALB/c nude mice were purchased from Beijing Vital River Laboratory Animal Technology Co., Ltd. (Beijing, China). All animals were kept in a specific pathogen-free environment in this study. 5×10^5^ MC38 cells were injected into the inguinal folds of C57BL/6 to construct the MC38 Syngeneic CRC mouse model. 1×10^6^ DLD-1 cells were injected into the inguinal folds of BALB/c to construct the DLD1 Xenograft CRC mouse model. These mice were sacrificed for collection blood at 28 days after injection. Besides, C57BL/6 mice were treated with azoxymethane (AOM) and dextran sodium sulfate (DSS) to construct AOM/DSS CRC model. C57BL/6 mice were injected intraperitoneally with 12.5mg/kg AOM, after which they were given 2.5% DSS in water for 1 week and then water only for 1 week. This cycle was repeated three times. AOM/DSS mice were sacrificed for collection blood at 6 weeks after injection. Apc-L850X mice, a model of spontaneous CRC, were purchased from Shanghai Model Organisms Center, Inc. Apc-L850X mice were sacrificed for collection blood at 14 weeks old. In MC38 Syngeneic group, Xenograft+Oxaliplatin group and Xenograft+5-FU groups, one mouse each had blood not collected because of coagulation. All procedures related to animal feeding, treatment, and welfare were conducted following with the Institutional Animal Care and Use Committee of Sun Yat-sen University.

### Cell lines and culture

The human CRC cell lines (SW480, RRID: CVCL_0546; SW620, RRID: CVCL_ 0547) and monocyte cell line (THP-1, RRID: CVCL_0006) were purchased from Celcook Biotech Co., Ltd. (Guangzhou, China). The mouse CRC cell line (MC38) was provided by Professor Zhengming Zhu, from the Seventh Affiliated Hospital of Sun Yat-Sen University. All human cell lines have been authenticated by Celcook Biotech Co., Ltd. (Guangzhou, China) and IGE Biotech Co., Ltd. (Guangzhou, China) using STR profiling within the last three years. All experiments were performed with mycoplasma-free cells. Cells were cultured in RPMI1640 and supplemented with 10% FBS at 37°C in a humidified incubator containing 5% CO2.

### RNA m^5^C quantification

Levels of m^5^C in total RNA were detected by MethyFlash 5-mC RNA Methylation ELISA Easy Kit (Fluorometric) (Epigentek, New York, USA). First, 200 ng RNA was added to assay wells covered with binding solution and incubated at 37°C for 90 minutes. Next, 5-mC antibody, signal indicator, and enhancer solution were sequentially added with diluted concentration and set at room temperature for 60 minutes. Lastly, added fluorescence development solution and incubated at room temperature for 3 minutes. Read the fluorescence on synergyH1 multi-modelreaders (BioTek, Vermont, USA) within 2 to 10 minutes at 530ex/590em nm.

### RNA isolation and qRT-PCR

Total RNA was extracted using TRIzol (Thermo Scientific, MA, USA). The qRT-PCR analysis system adopted SYBR Green Premix Pro Taq HS qPCR Kit (Accurate Biology, Changsha, China) and CFX96 Real-Time PCR Detection System (Bio-Rad, Shanghai, China). Moreover, GAPDH was used for normalization. As **Supplementary Table 2** shown, primers for related genes were listed.

### Bioinformatics analysis

The RNA-seq data for HC and CRC blood were taken from GEO databases (GSE10715). Differential expression analysis was conducted using R studio’s “limma” package (4.1.1) software. Gene set variation analysis (GSVA) was performed to evaluate m^5^C-modified pathways. Immune infiltration in blood was estimated by the MCP-Counter method.

### Statistical analysis

The data variability, which was presented as the SD (mean ± SD), was analyzed *via* unpaired Student’s *t* test between two groups for normally distributed data. Otherwise, the data was analyzed *via* nonparametric Mann–Whitney test. The effects of surgical resection treatment on m^5^C levels were analyzed *via* Paired t-tests. For multiple groups, significant differences were determined using one-way ANOVA. Discrimination was analyzed *via* the receiver operating characteristic (ROC) curve with an area under the curve (AUC). Reclassification was analyzed *via* the category-free net reclassification improvement (NRI) and integrated discrimination index (IDI). Construction of forest plot for multivariate logistic regression analysis to obtain diagnostic indexes for predicting CRC. Person correlation analysis was conducted to correlate GSVA scores, and immune infiltrates. *P*<0.05 was defined statistical significance.

## Results

### Levels of m^5^C in peripheral blood immune cells of CRC patients

**Figure 1:**
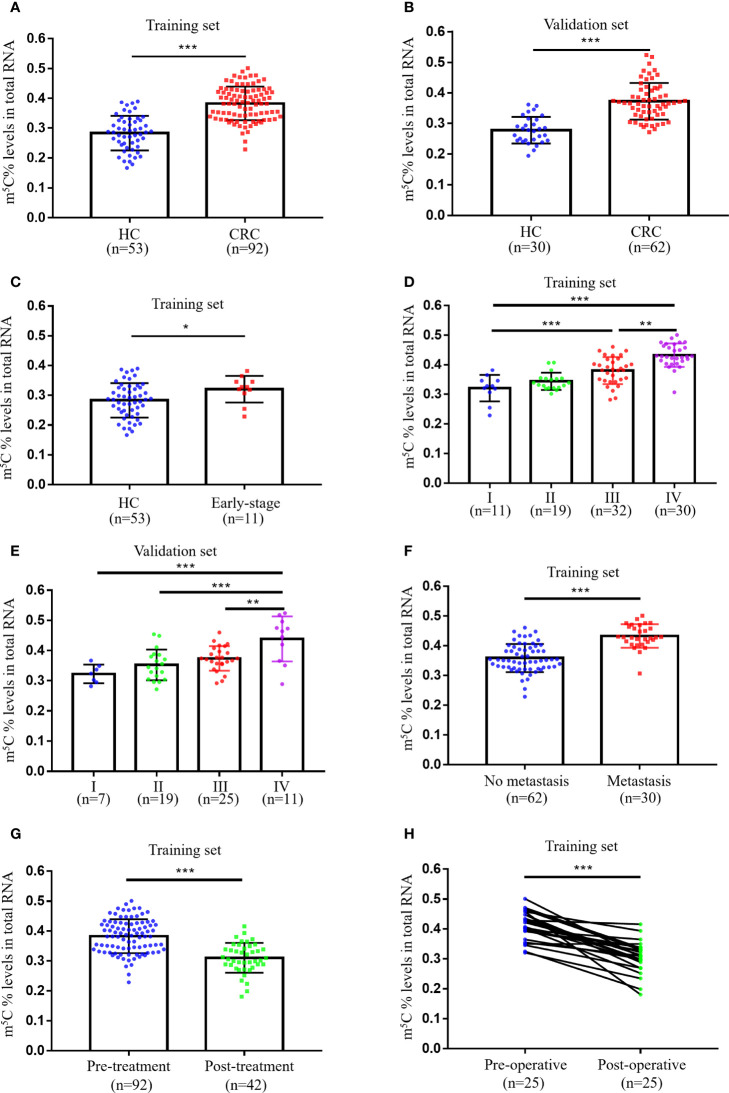
Levels of m^5^C in peripheral blood immune cells of CRC patients. **(A)** Levels of m^5^C in RNA of peripheral blood immune cells from HC (n=53) and CRC patients (n=92) in the training set. **(B)** Levels of m^5^C in RNA of peripheral blood immune cells from HC (n=40) and CRC patients (n=52) in the validation set. **(C)** Comparison of m^5^C levels in blood immune cells of HC (n=53) and early-stage CRC patients (n=11) in the training set. **(D)** m^5^C levels of blood immune cells in CRC patients at different clinical stages (stage-I, n=11; stage-II, n=19; stage-III, n=32; stage-IV, n=30) in the training set. **(E)** m^5^C levels of blood immune cells in CRC patients at different clinical stages (stage-I, n=7; stage-II, n=19; stage-III, n=25; stage-IV, n=11) in the validation set. **(F)** Comparison of blood m^5^C levels in CRC patients with (n=30) and without (n=62) distant-metastasis in the training set. **(G)** Comparison of blood m^5^C levels in CRC patients with (n=42) and without (n=92) treatment in the training set. **(H)** Levels of m^5^C in blood immune cells of CRC patients (n=25) before and after surgical resection treatment in the training set. Data are shown as mean ± SD; *P < 0.05, **P < 0.01 and ***P < 0.001.

Initially, we collected total RNA from peripheral blood immune cells of 53 healthy individuals and 92 untreated CRC patients to assess the status of m^5^C modification. Compared with healthy controls (0.283 ± 0.058), m^5^C levels in peripheral blood immune cells of CRC patients were significantly elevated in the training set (0.383 ± 0.057; **Figure 1A**). At the same time, m^5^C levels were found to be significantly higher in the validation set (0.373 ± 0.060; **Figure 1B**). Impressively, increased m^5^C levels were also observed in early-stage CRC patients that were difficult to diagnose by non-invasive methods in both training set and validation set (**Figure 1C**; **Supplementary Figure 1A**). Furthermore, the analysis results of m^5^C levels and clinicopathological characteristics of CRC patients revealed that levels of m^5^C were related to clinical stage, N classification, and M classification (**Table 1**; **Supplementary Table 1**). Consistent with the results in the training set, m^5^C levels in CRC patients’ blood gradually increased as stage progression in the validation set (**Figures 1D, E**). Compared with patients without distant-metastasis, m^5^C levels were markedly enhanced in CRC patients with distant-metastasis (**Figure 1F**; **Supplementary Figure 1B**). Our data also showed that m^5^C levels in peripheral blood were positively with m^5^C levels in the corresponding CRC tumor tissue. (**Supplementary Figure 1C**). Overall, these results demonstrated that m^5^C levels in peripheral blood immune cells were dramatically raised in CRC patients and increased with tumor stage.

Currently commonly used blood tumor biomarkers have been reported to monitor the therapeutic status of oncology patients, we thus evaluate whether m^5^C has a similar function. Our results indicated that m^5^C levels in blood were markedly reduced in the Post-treatment group (0.321 ± 0.045; **Figure 1G**). In addition, we examined changes in m^5^C levels at admission and 14 days after surgery in 25 CRC patients, suggesting a significant decrease in m^5^C levels after treatment (**Figure 1H**). Taken together, m^5^C modification in peripheral blood immune cells might be a biomarker for CRC surveillance.

### Clinical utility of m^5^C modification in peripheral blood immune cells for the diagnosis of CRC

**Figure 2:**
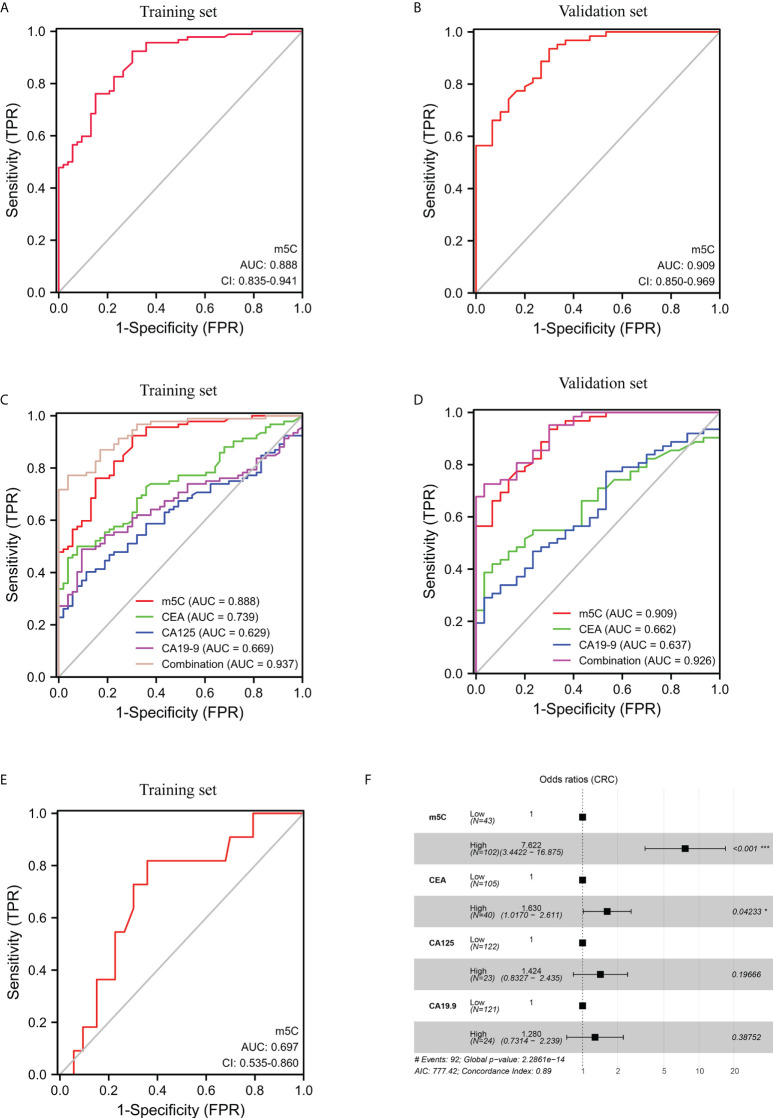
Clinical utility of m^5^C modification in peripheral blood immune cells for the diagnosis of CRC. **(A, B)** ROC curve for m^5^C modification of peripheral blood immune cells in the training set **(A)** and validation set **(B)**. **(C, D)** ROC curve for m^5^C modification compared with CEA, CA19-9, and CA125 in the training set **(C)** and validation set **(D)**. **(E)** ROC curve for m^5^C modification of early-stage CRC in the training set. **(F)** Forest plot of multivariate logistic regression indicated predictive factors for CRC diagnosis in the training set.

The efficacy of m^5^C modification in peripheral blood immune cells for the diagnosis of CRC was assessed by means of discrimination and reclassification. Discrimination performance was evaluated by plotting the ROC curve and thus calculating AUC value. In the training set, the AUC of m^5^C modification in peripheral blood immune cells was as high as 0.888 (95% CI, 0.835-0.941; **Figure 2A**). While the AUC of m^5^C modification in the validation set was 0.909 (95% CI, 0.850-0.969), suggesting this biomarker could distinguish CRC patients from healthy controls (**Figure 2B**). In addition, the optimal cutoff value of m^5^C was 0.311 in the training set and 0.294 in the validation set (**Supplementary Figures 2A, B**). Notably, levels of m^5^C in peripheral blood immune cells had better discrimination ability than conventional serological biomarkers such as CEA, CA19-9, and CA125, with AUCs of 0.739, 0.669, and 0.629, in the training set (**Figure 2C**; **Table 2**). Furthermore, The AUC for the multivariate combination of m^5^C, CEA, CA19-9, and CA125 improved to 0.937 (95% CI, 0.901-0.973; **Figure 2C**). Similarly, m^5^C modification had a higher AUC value than CEA and CA19-9 in the validation set (**Figure 2D**; **Supplementary Table 3**). Levels of m^5^C also presented good discrimination ability in stage-I CRC with AUCs of 0.697 and 0.795 in the training set and validation set, respectively (**Figure 2E**; **Supplementary Figure 2C**). Furthermore, using NRI and IDI to evaluate the performance of reclassification. The NRI of m^5^C compared to CEA, CA19-9, and CA125 were 0.461, 0.706, and 0.750, while the IDI of m^5^C compared to CEA, CA19-9, and CA125 were 0.241, 0.290, and 0.320 (**Table 3**). These results implied that m^5^C levels had superior reclassification performance than common CRC blood biomarkers in CRC diagnosis. Besides, a forest plot of multivariate logistic regression was constructed along with common tumor biomarkers in the training set (**Figure 2F**). The results suggested that m^5^C and CEA were independent risk factors related to CRC diagnosis, with m^5^C modification displaying the highest odds ratio of being diagnosed as CRC (**Figure 2F**, odds ratio=7.622). Therefore, these results revealed that m^5^C modification in blood was a valuable biomarker for the diagnosis of CRC.

**Table 3:** NRI and IDI of m^5^C compared with other tumor biomarkers.

	m^5^C	CEA	CA125	CA19-9
NRI	Reference	0.461 (0.141-0.782)	0.750 (0.436-1.064)	0.706 (0.390-1.022)
IDI	Reference	0.241 (0.139-0.343)	0.320 (0.220-0.420)	0.290 (0.191-0.390)

**Table 2:** Sensitivity and specificity of the diagnostic value of tumor biomarkers in the training set.

Marker	Sensitivity	Specificity	AUC	95% CI
m^5^C	0.924	0.698	0.888	0.835-0.941
CEA	0.500	0.925	0.739	0.660-0.818
CA125	0.402	0.887	0.629	0.540-0.718
CA19-9	0.489	0.906	0.669	0.583-0.755
m^5^C+CEA+CA125+CA19-9	0.772	0.962	0.937	0.901-0.973

### Levels of m^5^C in peripheral blood immune cells of CRC tumor-bearing mice

**Figure 3:**
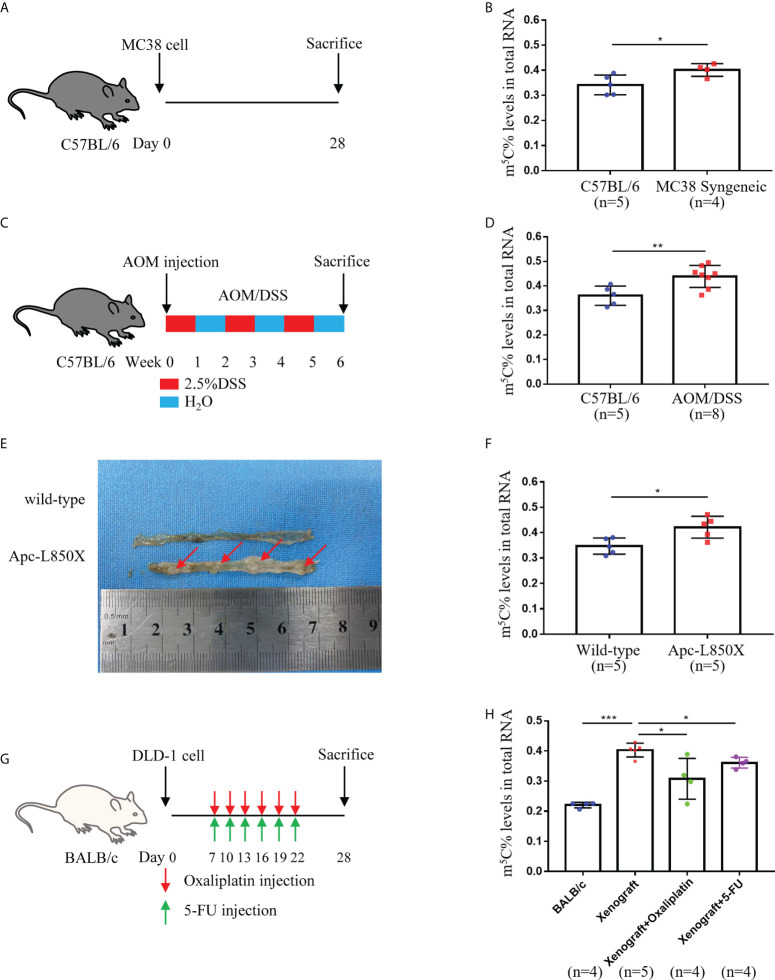
Levels of m^5^C in peripheral blood immune cells of CRC tumor-bearing mice. **(A)** Schematic representation of the MC38 Syngeneic CRC mouse model. **(B)** Comparison of m^5^C levels in blood immune cells between C57BL/6 controls and MC38 Syngeneic mice. **(C)** Schematic representation of the AOM/DSS CRC mouse model. **(D)** Comparison of m^5^C levels in blood immune cells between C57BL/6 controls and AOM/DSS mice. **(E)** Representative tumor images of the colon in Apc-L850X mice were shown. **(F)** Comparison of m^5^C levels in blood immune cells between wild-type mice and Apc-L850X mice. **(G)** Schematic representation of the DLD-1 Xenograft CRC mouse model with Oxaliplatin or 5-FU treatment. **(H)** Comparison of m^5^C levels in blood immune cells between untreated and treated DLD-1 Xenograft mice. Data are shown as mean ± SD; *P < 0.05, **P < 0.01, ***P < 0.001.

To further verify whether CRC tumor cells could increase m^5^C levels in peripheral blood immune cells *in vivo*, we collected blood from a series of CRC mouse models. 28 days after subcutaneous injection of tumor cells, increased m^5^C levels were detected in MC38 Syngeneic mice compared with C57BL/6 controls (**Figures 3A, B**; **Supplementary Figure 3A**). Constructing primary CRC mice with the chemical inducer AOM/DSS, we found that the blood of AOM/DSS mice also had a dramatic rise in m^5^C levels (**Figures 3C, D**). By replacing the amino acid L at position 850 of the Apc gene with an X, a mouse model of a point mutation in the Apc gene was established, which causes multiple adenomas in the colon (**Figure 3E**; **Supplementary Figure 3B**). The results presented that Apc-L850X mice possessed higher levels of m^5^C than wild-type mice (**Figure 3F**). Next, we constructed a DLD-1 Xenograft mouse model to assess whether peripheral blood m^5^C levels could also monitor the therapeutic status of tumor-bearing mice (**Figure 3G**). Compared with non-implanted BALB/c mice, m^5^C levels were significantly higher in peripheral blood of DLD-1 Xenograft mice (**Figure 3H**). Interestingly, similar to the results in CRC patients, levels of m^5^C were reduced in DLD-1 Xenograft mice after treatment with 5-FU or oxaliplatin (**Figure 3H**; **Supplementary Figure 3C**). Overall, increased levels of m^5^C were observed in peripheral blood immune cells of CRC tumor-bearing mice.

### Expressions of m^5^C-modified regulators NSUN5 and YBX1 in peripheral blood immune cells of CRC

**Figure 4:**
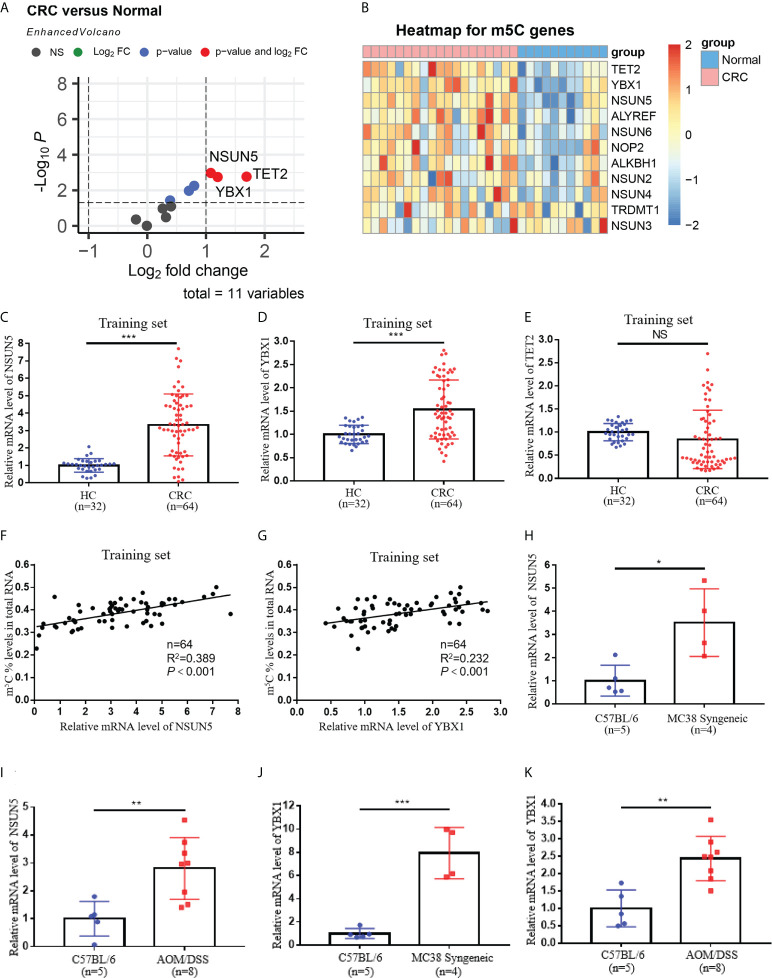
Expressions of m^5^C-modified regulators NSUN5 and YBX1 in peripheral blood immune cells of CRC. **(A)** Screening of key molecules associated with m^5^C modification in peripheral blood immune cells of CRC patients by limma differential analysis. **(B)** Heatmap of key molecules associated with m^5^C modification in blood immune cells of CRC patients. **(C-E)** qRT-PCR analysis of NSUN5**(C)**, YBX1 **(D)**, and TET2 **(E)** mRNA expression levels in blood immune cells of HC and CRC patients in the training set. **(F)** Correlation between the levels of NSUN5 and m^5^C in blood immune cells of CRC patients in the training set. **(G)** Correlation between the levels of YBX1 and m^5^C in blood immune cells of CRC patients in the training set. **(H, I)** qRT-PCR analysis of NSUN5 mRNA expression levels in blood immune cells of MC38 Syngeneic mice**(H)** and AOM/DSS mice**(I)**. **(J, K)** qRT-PCR analysis of YBX1 mRNA expression levels in peripheral blood immune cells of MC38 Syngeneic mice **(J)** and AOM/DSS mice**(K)**. Data are shown as mean ± SD; *P < 0.05, **P < 0.01, ***P < 0.001.

To investigate the causes of elevated m^5^C levels in peripheral blood immune cells of CRC patients, we used bioinformatics methods to analyze the expression of relevant readers, erasers, and writers that regulate m^5^C modifications in the GSE10715 dataset (**Figures 4A, B**). Among these regulators, NSUN5, YBX1, and TET2 were elevated in blood immune cells of CRC patients by limma differential analysis (**Figures 4A, B**). Further qRT-PCR assays performed in training set and validation set samples revealed that only NSUN5 and YBX1 were enhanced in blood of CRC patients, whereas TET2 was not significantly altered (**Figures 4C-E**; **Supplementary Figure 4A, B**). We also found that m^5^C levels correlated with NSUN5 and YBX1 expression but not TET2 expression in both training set and validation set (**Figures 4F, G**; **Supplementary Figures 4C-E**). qRT-PCR results demonstrated elevated levels of NSUN5 and YBX1 in blood immune cells of CRC tumor-bearing mice (**Figures 4H-K**). Collectively, m^5^C-modified regulators NSUN5 and YBX1 were identified to be responsible for elevated m^5^C levels in blood immune cells of CRC patients.

### m^5^C modification of monocyte in peripheral blood of CRC patients.

**Figure 5:**
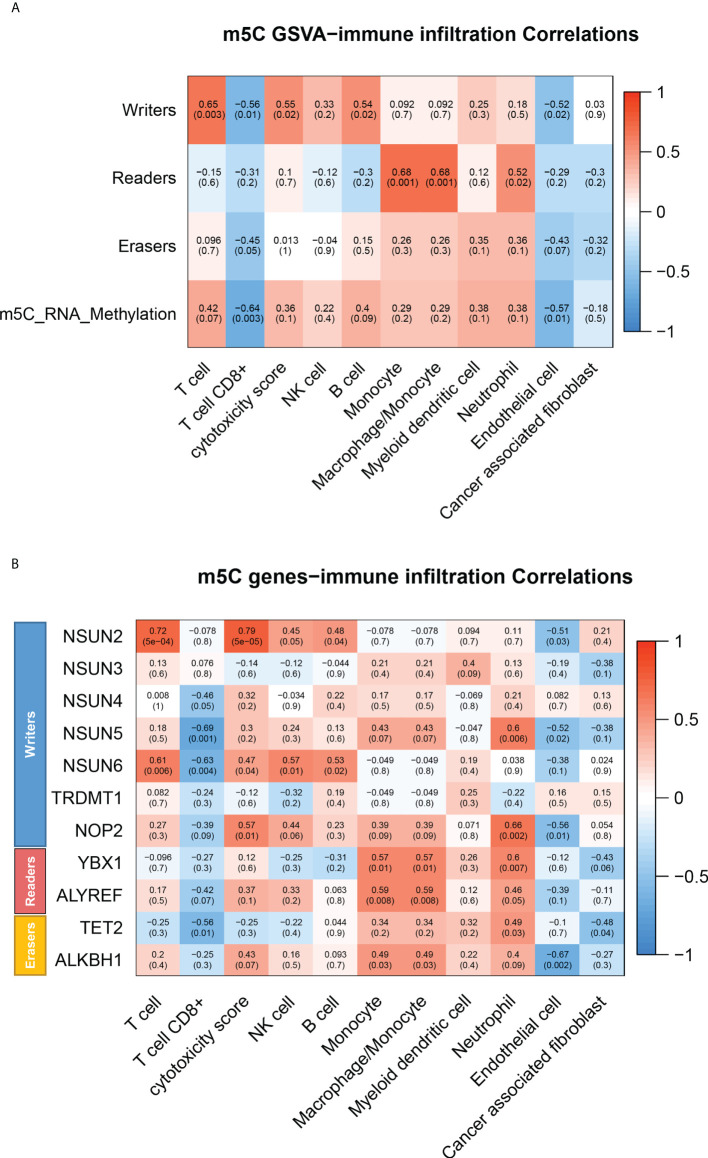
m^5^C modification of various immune cells in peripheral blood of CRC patients. **(A)** Heatmap of correlation between infiltrating immune cell types and m^5^C modification pathways in blood of CRC patients by GSVA. **(B)** Heatmap of correlation between infiltrating immune cell types and m^5^C modification related gene in blood of CRC patients by GSVA. NS, no significance.

To further define which type of immune cell have elevated m^5^C levels in peripheral blood of CRC patients, GSVA was performed to assess the correlation of m^5^C modification-related pathways with various immune cells infiltration based on GSE10715. The results showed that the m^5^C methyltransferase complex, made up of writers, readers, and erasers, presented the strongest positive association with monocyte infiltrating in blood of CRC patients (**Figure 5A**). Detection of monocytes and non- monocytes immune cells isolated from peripheral blood of CRC patients also indicated that monocytes had higher m^5^C levels (**Supplementary Figure 4F**). Meanwhile, Heatmap revealed that infiltration of monocytes in CRC patients’ blood was distinctly associated with the expression of NSUN5 and YBX1, Consistent with the important roles of NSUN5 and YBX1 in m^5^C remodeling found in **Figure 4** (**Figure 5B**). Taken together, monocyte was the predominant m^5^C-modified immune cell type in blood of patients with CRC.

**Figure 6:**
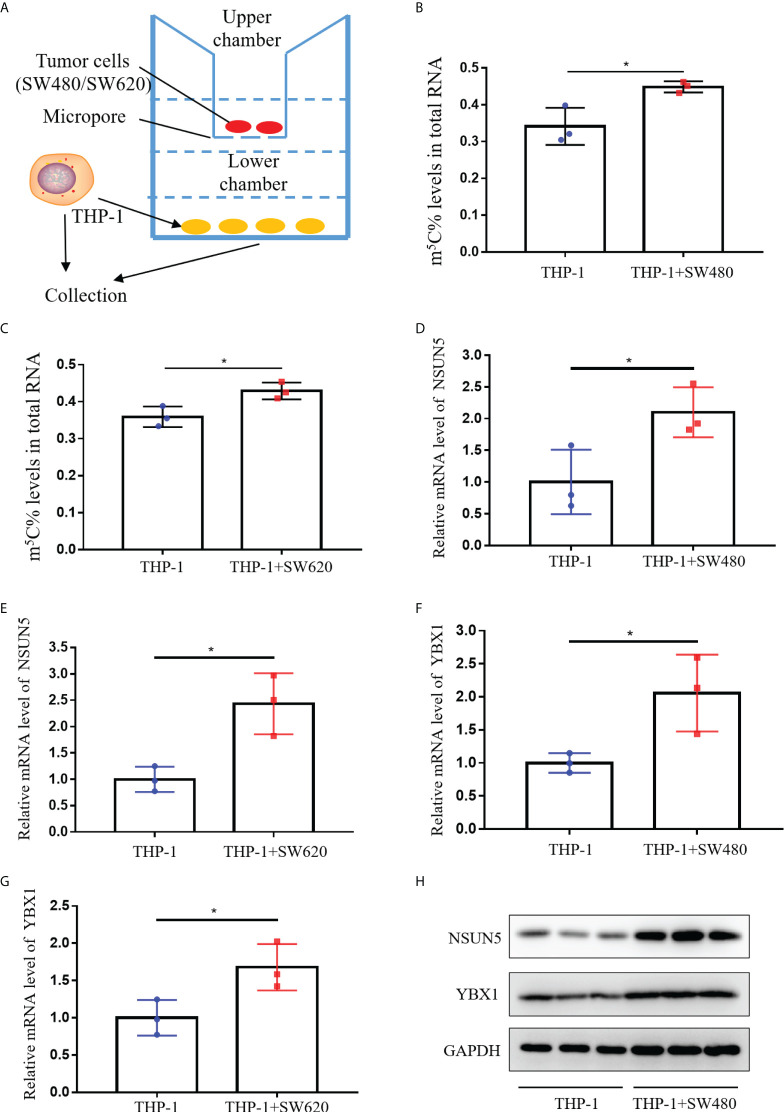
Levels of m^5^C and regulator in monocyte co-culture with CRC cells. **(A)** Schematic representation of the co-culture model of THP-1 and CRC cells. **(B)** Comparison of m^5^C levels between THP-1 with and without SW480 co-culture. **(C)** Comparison of m^5^C levels between THP-1 with and without SW620 co-culture. **(D, E)** qRT-PCR analysis of NSUN5 mRNA expression levels in THP-1 with SW480 **(D)** or SW620 **(E)** co-culture. **(F, G)** qRT-PCR analysis of YBX1 mRNA expression levels in THP-1 with SW480 **(F)** or SW620 **(G)** co-culture. **(H)** Western blot analysis of NSUN5 and YBX1 protein expression in THP-1 with or without SW480 co-culture. Data are shown as mean ± SD; *P < 0.05.

To assess whether CRC cells could enhance m^5^C levels of monocyte *in vitro*, we examined m^5^C levels in THP-1 cells after co-culture with SW480 or SW620 cells. As shown in **Figure 6A**, SW480 or SW620 cells were seeded in the upper chamber of transwell, while THP-1 cells were seeded in the lower chamber of transwell. After co-culture with SW480 or SW620 cells for 48 hours, m^5^C levels of THP-1 cells were dramatically increased (**Figures 6B, C**). Meanwhile, the qRT-PCR results showed that the mRNA levels of both NSUN5 and YBX1 were markedly elevated in THP-1 cells co-culture with SW480 or SW620 cells (**Figures 6D-G**). Furthermore, western blot analysis showed enhanced protein expression of NSUN5 and YBX1 in THP-1 co-cultured with SW480 or SW620 (**Figure 6H**; **Supplementary Figure 4G**). Overall, our results demonstrated that levels of m^5^C and related regulators were all raised in monocytes co-cultured with CRC cells.

## Discussion

The majority of patients are diagnosed with CRC at an advanced stage, which leads to a poor prognosis (1). Hence, improving CRC patients’ prognosis relies on a simple and precise diagnosis. Peripheral blood was widely utilized for tumor diagnosis because of its high content of cellular metabolites and easy access from patients (21). Alternatively, the present blood tumor biomarkers of CRC have insufficient sensitivity to diagnose CRC (5). The liquid biopsy technology represented by exosomes was still in the research stage, and the complicated information accompanying deep sequencing made it challenging to analyze the subsequent results (22). Consequently, using other validated biomarkers to optimize the diagnosis of CRC was essential. The presence of many leukocytes in peripheral blood, which might carry genetic information related to tumor cells, has been neglected in the diagnosis of tumors. This research determined that m^5^C modification in blood immune cells was a potential biomarker for CRC screening.

Our recent study provided preliminary evidence that m^6^A methylation modification of mRNA could be used as a marker for CRC diagnosis (14). m^5^C was also another primary modality of mRNA methylation modification, but investigations conducted in m^5^C were not as intensive as m^6^A due to the lack of reliable detection methods. Previous article reported that m^5^C-modified regulators were significantly elevated in tumor tissue of gastric cancer (23), pancreatic cancer (24), breast Cancer (25), and leukemia (26). With the advent of m^5^C assay kits, we decided to further explore the modification status of m^5^C in CRC patients’ blood. The first findings of our study indicated that m^5^C levels of RNA in peripheral blood immune cells of CRC patients were substantially increased than those of healthy individuals (**Figures 1A, B**). Moreover, m^5^C contents of blood immune cells gradually raised as stage progression (**Figures 1D, E**). Interestingly, these results were in line with the conclusion that the expression of m^5^C signatures in CRC tissue was related to different clinical outcomes and tumor status found by other researchers (19). It might be since with the development of the stage, more tumor cells were released into the blood during epithelial-mesenchymal transition (EMT), affecting the phenotype of immune cells (27–29). Unfortunately, the samples collected in our study were within the past two years, and prognostic data are currently unavailable. We would continue to monitor these patients to observe the relationship between m^5^C levels and prognosis. Given the high recurrence rate of CRC, it would be vital to explore the relationship between m^5^C and tumor recurrence once we received follow-up data. Furthermore, m^5^C contents were decreased in treated CRC patients or mice, suggesting that it might be an index for monitoring treatment status (**Figures 1G, H**; **Figures 3G, H**). Nevertheless, additional clinical samples needed to be collected for determining its potential as an index of efficacy, such as tumor recurrence and drug resistance.

The blood tumor biomarkers CEA, CA19-9, and CA125, were broadly employed for physical screening of CRC (7). However, these three indicators were more appropriate for postoperative risk monitoring in CRC patients due to their lower sensitivity (30). Our results displayed that m^5^C modification discriminated between CRC patients and healthy recipients with an AUC of 0.888 (95% CI, 0.835-0.941), was substantially superior to that of CEA (0.739; 95% CI, 0.660-0.818), CA19-9 (0.669; 95% CI, 0.583-0.755), and CA125 (0.629; 95% CI, 0.540-0.718) for AUC in the training set (**Figure 2C**). These results are consistent with those in the validation set (**Figure 2D**). The coupling of CEA, CA19-9, and CA125 with m^5^C raised the AUC to 0.937 (95% CI, 0.901-0.973), suggesting that the combination would confer a better discrimination performance (**Figure 2C**). Our limited sample of early-stage CRC patients indicated a rise in m^5^C levels and an AUC of 0.697 in the training set (**Figure 1C**; **Figure 2E**). Although these results were also observed in the validation set, the availability of m^5^C for early-stage CRC screening required further studies to evaluate (**Supplementary Figures 1A**, **2C**). Taken together, more cohort data were necessary to verify the diagnostic value of m^5^C modification before applying it to clinical detection.

In recent literature, the fecal immunochemical test (FIT), DNA mutation, DNA methylation, and microbial dysbiosis all showed promising in CRC non-invasive detection. FIT tested gastrointestinal bleeding by detecting hemoglobin in the stool. Gastrointestinal bleeding was one of the signs of CRC, about 20% of patients with early stage CRC were FIT positive, and 90% of patients with advanced CRC were FIT positive (31). Gastrointestinal hemorrhage was not only a specific symptom of CRC patients, but also a variety of common digestive tract inflammatory diseases were often accompanied by FIT positive, which could not differentiate between CRC and benign diseases causing gastrointestinal bleeding (32). Methylation of DNA in cancer tended to occur at thousands of CpG sites, making it easier to detect and assess (33). Simultaneous methylation patterns could reflect the epigenetic origin of specific cancers and were used to reveal the tissue of origin of unknown primary cancers (34). Therefore, the detection of DNA methylation was stronger than the detection of DNA mutation in both sensitivity and location of cancer (34). However, the current DNA methylation detection technology had extremely high DNA damage, which could cause about 90% of the DNA template to be lost (35). Although DNA methylation sequencing could be optimized through a series of primer design, library optimization and other methods, there were still major shortcomings in clinical application. Reprogramming of gut microbiota in CRC patients correlated with changes in serum metabolome, and gut microbiome-associated serum metabolites had potential applications in detecting CRC and adenomas (36). However, the microbial metabolomic assays were expensive and could not currently be the main method for large-scale screening of CRC. Compared with the above-mentioned techniques, peripheral blood immune cell samples were easily obtained, and m^5^C methylation assays were inexpensive. Therefore, m^5^C methylation of peripheral blood immune cells could be used as a simple and feasible noninvasive diagnostic biomarker for CRC screening.

m^5^C-modified regulators NSUN5 and YBX1 were screened out to be responsible for elevated m^5^C levels through bioinformatics analysis and qRT-PCR validation (**Figure 4**). NSUN5, an m^5^C methyltransferase belonged to NSUN family, was identified as a promoter in CRC progression *via* cell cycle regulation (37). One study reported that YBX1, the “readers” of m^5^C modification, activated NF signaling pathway in CRC (38). Moreover, the transcription factor YBX1 enhanced the expression of NRF2 by binding to its promoter region, promoting the proliferation of CRC cells (39). YBX1 also served as a mediator of signaling in the EGFR-RAS-MAPK axis (40). Current studies have identified the carcinogenic role of NSUN5 and YBX1 in CRC tumor cells, but their expression and function in the immune microenvironment remained unclear. Our results revealed elevated terms of NSUN5 and YBX1 in blood of CRC patients and CRC mouse models (**Figure 4**). *In vitro* co-culture experiments also demonstrated that CRC tumor cells promoted NSUN5 and YBX1 expression in immune cells, resulting in elevated m^5^C levels (**Figure 6**). The ensuing question was how CRC tumor cells led to high expression of the m^5^C-modified regulators NSUN5 and YBX1 in immune cells of peripheral blood. A large number of extracellular vesicles secreted by CRC tumor cells were present in blood (41). Growing evidence implied that extracellular vesicles derived from CRC tumor cells were absorbed by monocytes to regulate their phenotype and cytokine lineage (42). Analysis of the exosome database revealed that peripheral blood extracellular vesicles from CRC patients contained more YBX1 (data not shown). However, the expression of NSUN5 in extracellular vesicles was not visibly elevated, suggesting that multiple complex mechanisms might be involved (data not shown).

The bioinformatics results suggested that monocyte was the predominant m^5^C-modified immune cell type in blood of CRC patients (**Figure 5**). Tumor-educated circulating monocytes were powerful candidate biomarkers for the diagnosis and monitoring of CRC (43). Interestingly, our previous study also found monocyte was the most strongly m^6^A-modified immune cells in CRC patients’ blood (14). Non-coincidentally, other research reported that higher proportion of promoter methylation of NDRG4 and TFPI2 genes in monocyte was associated with a high stage of CRC (44). These results implied that monocyte was closely associated with methylation modifications and exert vital functions in tumor diagnosis. The nucleocapsids of SARS-CoV-2 were also detected in blood monocyte of COVID-19 patients, indicating that SARS-CoV-2 might infect monocyte (45–47). Meanwhile, monocytes in blood of COVID-19 patients were in the activated state of pyroptosis (48). Pyroptosis-induced monocyte death and subsequent released of proinflammatory cytokines might be a reason for the poor prognosis of COVID-19 patients (48). Whether monocyte subpopulations with increased m^5^C levels performed a similar function in tumors warrants further exploration.

In conclusion, the highlight of our research is the confirmation that m^5^C modification in peripheral blood immune cells of CRC patients can be utilized as a promising non-invasive diagnostic biomarker. Besides, m^5^C-modified regulators NSUN5 and YBX1 are identified to be responsible for the elevated m^5^C levels. Monocytes are the predominant m^5^C-modified immune cell type in blood of CRC patients.

## Data availability statement

The original contributions presented in the study are included in the article/**Supplementary Material**. Further inquiries can be directed to the corresponding authors.

## Ethics statement

The studies involving human participants were reviewed and approved by the Ethics Committee of the Zhongshan People’s Hospital and Sun Yat-sen University Cancer Center. The patients/participants provided their written informed consent to participate in this study. The animal study was reviewed and approved by Institutional Animal Care and Use Committee of Sun Yat-sen University.

## Author contributions

HY, ZH, NS, and ML performed the experiments and analyzed the data. CZ, YH, and XJ conceived and designed this study. GL, JH, and HW contributed to the reagent preparation and subject discussion. All authors contributed to the article and approved the submitted version.

## Funding

This study was supported by the National Natural Science Foundation of China (81902693; 81901557; 82073148), the Sanming Project of Medicine in Shenzhen (SZSM201911010), the Shenzhen Sustainable Project (KCXFZ202002011010593), the Shenzhen Key Medical Discipline Construction Fund (SZXK016), and the Guangdong Provincial Key Laboratory of Digestive Cancer Research (2021B1212040006).

## Conflict of interest

The authors declare that the research was conducted in the absence of any commercial or financial relationships that could be construed as a potential conflict of interest.

The handling editor GW declared a shared parent affiliation with the authors HY, ZH, SN, LM, HJ, LG, WH, YH, CZ at the time of review.

## Publisher’s note

All claims expressed in this article are solely those of the authors and do not necessarily represent those of their affiliated organizations, or those of the publisher, the editors and the reviewers. Any product that may be evaluated in this article, or claim that may be made by its manufacturer, is not guaranteed or endorsed by the publisher.

## Supplementary material

Supplementary Figure 1 Levels of m^5^C in peripheral blood immune cells of CRC patients in the validation set. **(A)** Comparison of m^5^C levels in blood immune cells of HC (n=30) and early-stage CRC patients (n=7) in the validation set. **(B)** Comparison of blood m^5^C levels in CRC patients with (n=11) and without (n=51) distant-metastasis in the validation set. **(C)** Correlation between m^5^C levels in peripheral blood and m^5^C levels in the corresponding CRC tumor tissue.

Supplementary Figure 2 Clinical utility of m^5^C modification for the diagnosis of CRC in the validation set. **(A, B)** Cutoff value for m^5^C modification of peripheral blood immune cells in the training set **(A)** and validation set **(B)**. **(C)** ROC curve for m^5^C modification of early-stage CRC in the validation set.

Supplementary Figure 3 The representative morphology and tumor weight **(A)** Representative tumor morphology of the colon from AOM/DSS mice were shown. **(B)** Tumors number of AOM/DSS mice and Apc-L850X mice. **(C)** Tumor weight of MC38 Syngeneic mice and DLD-1 Xenograft mice.

Supplementary Figure 4 Expressions of m^5^C-modified regulators NSUN5 and YBX1 in peripheral blood immune cells of the validation set. **(A, B)** qRT-PCR analysis of NSUN5 **(A)** and YBX1 **(B)** mRNA expression levels in blood immune cells of HC and CRC patients in the validation set. **(C)** Correlation between the levels of TET2 and m^5^C in the training set. **(D)** Correlation between the levels of NSUN5 and m^5^C in the validation set. **(E)** Correlation between the levels of YBX1 and m^5^C in the validation set. **(F)** m^5^C levels in monocytes from CRC patients (n=12) were higher than those in non-monocyte immune cells (n=12). **(G)** Western blot analysis of NSUN5 and YBX1 protein expression in THP-1 with or without SW620 co-culture.
