# Supplementary figures and images for: 5-Methylcytosine (m5C) modification in peripheral blood immune cells is a novel non-invasive biomarker for colorectal cancer diagnosis
Source: Front Immunol. 2022 Sep 21;13:967921. doi: 10.3389/fimmu.2022.967921 (PMC9532581; doi:10.3389/fimmu.2022.967921)

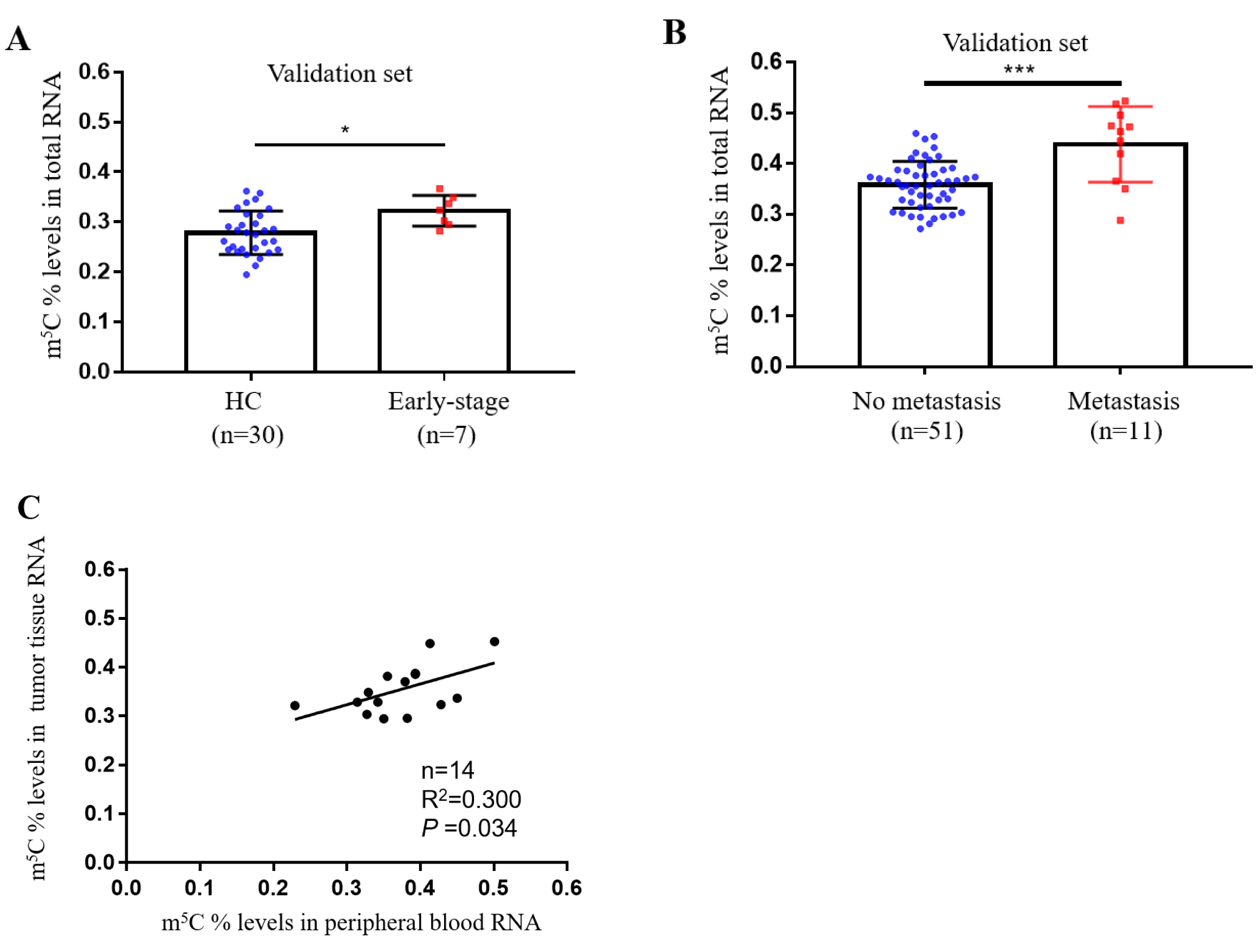

Supplement: Supplementary Figure 1 — Levels of m5C in peripheral blood immune cells of CRC patients in the validation set. (A) Comparison of m5C levels in blood immune cells of HC (n=30) and early-stage CRC patients (n=7) in the validation set. (B) Comparison of blood m5C levels in CRC patients with (n=11) and without (n=51) distant-metastasis in the validation set. (C) Correlation between m5C levels in peripheral blood and m5C levels in the corresponding CRC tumor tissue. [file Image_1.tif]

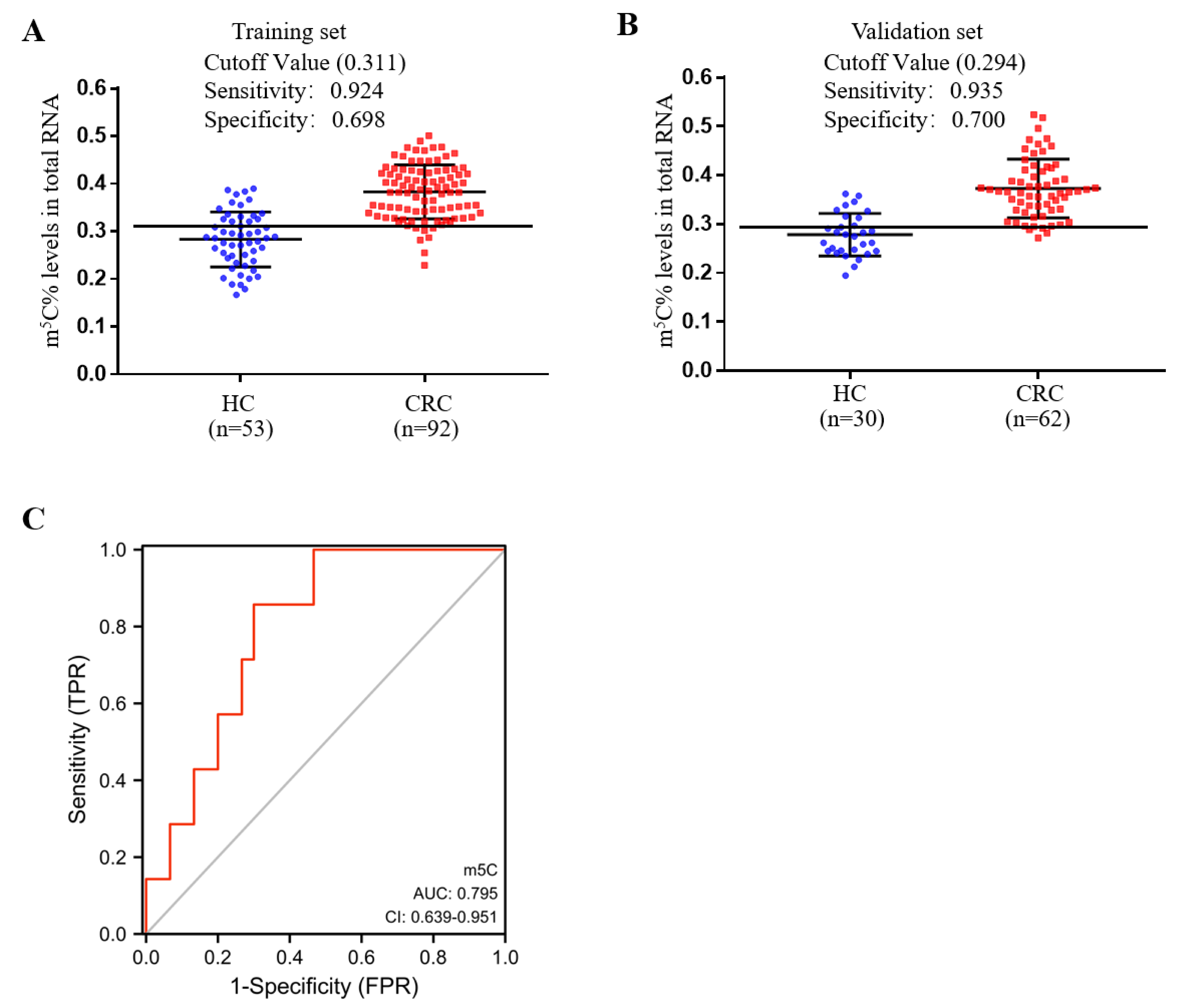

Supplement: Supplementary Figure 2 — Clinical utility of m5C modification for the diagnosis of CRC in the validation set. (A, B) Cutoff value for m5C modification of peripheral blood immune cells in the training set (A) and validation set (B). (C) ROC curve for m5C modification of early-stage CRC in the validation set. [file Image_2.tif]

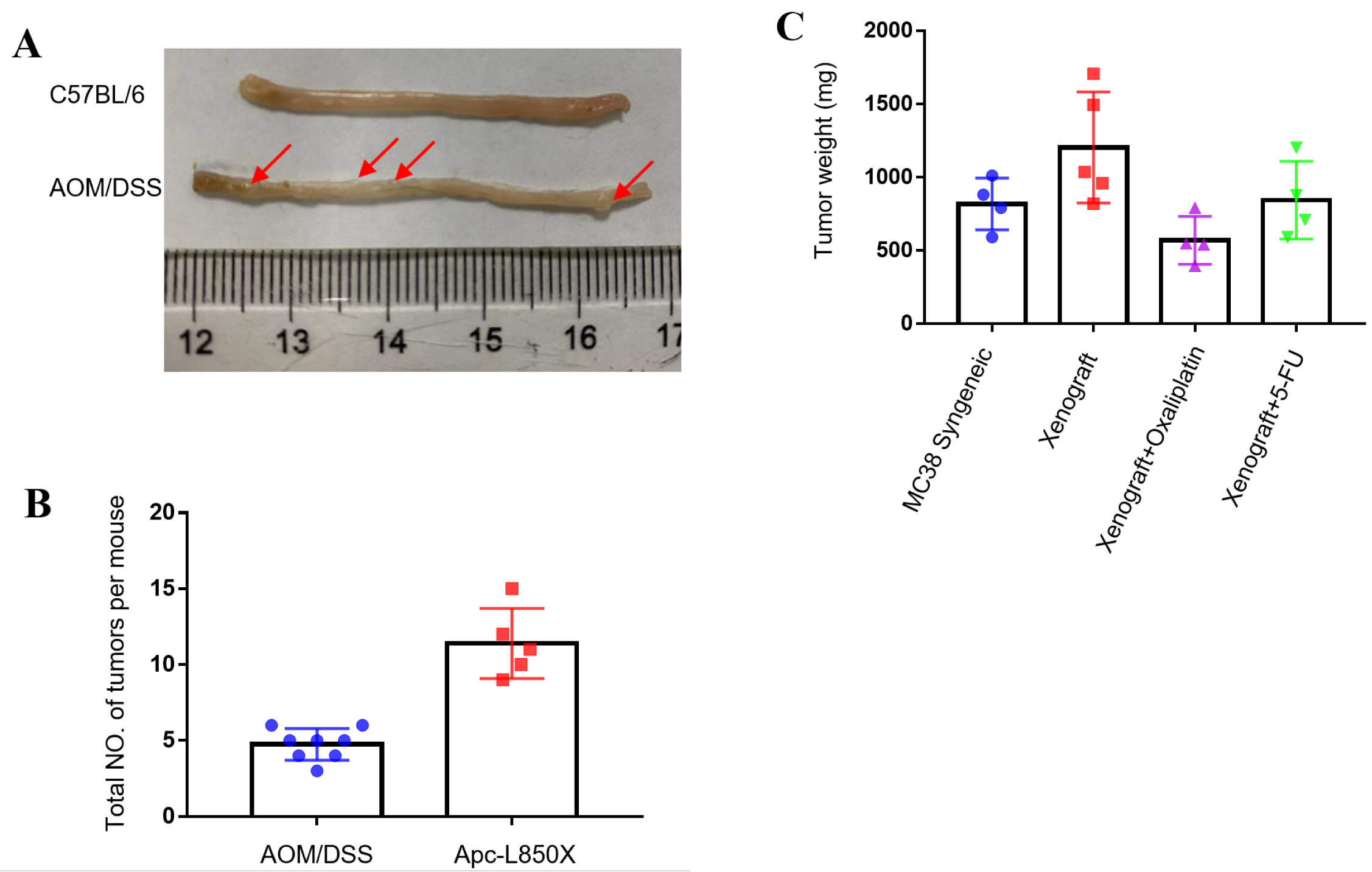

Supplement: Supplementary Figure 3 — The representative morphology and tumor weight (A) Representative tumor morphology of the colon from AOM/DSS mice were shown. (B) Tumors number of AOM/DSS mice and Apc-L850X mice. (C) Tumor weight of MC38 Syngeneic mice and DLD-1 Xenograft mice. [file Image_3.tif]

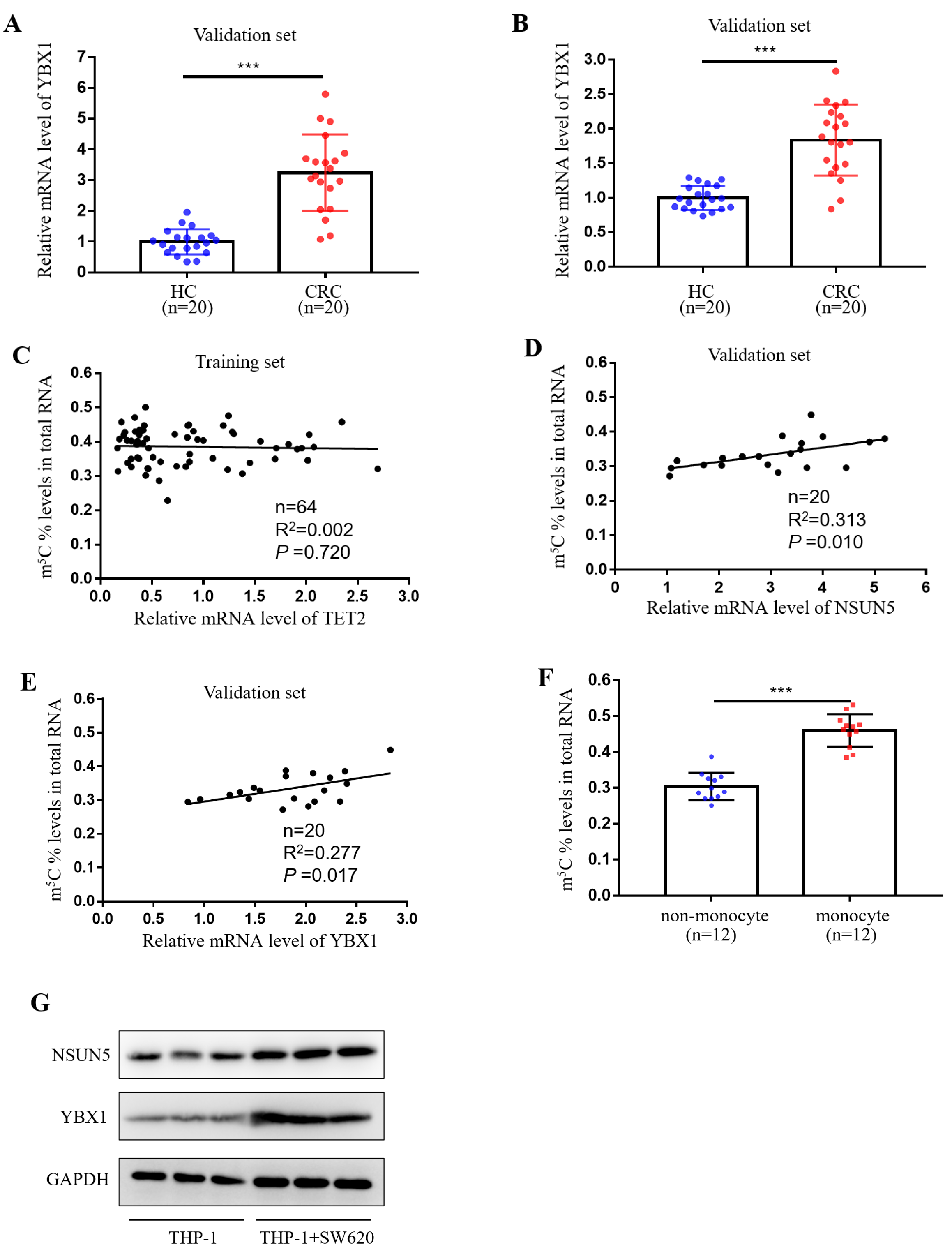

Supplement: Supplementary Figure 4 — Expressions of m5C-modified regulators NSUN5 and YBX1 in peripheral blood immune cells of the validation set. (A, B) qRT-PCR analysis of NSUN5 (A) and YBX1 (B) mRNA expression levels in blood immune cells of HC and CRC patients in the validation set. (C) Correlation between the levels of TET2 and m5C in the training set. (D) Correlation between the levels of NSUN5 and m5C in the validation set. (E) Correlation between the levels of YBX1 and m5C in the validation set. (F) m5C levels in monocytes from CRC patients (n=12) were higher than those in non-monocyte immune cells (n=12). (G) Western blot analysis of NSUN5 and YBX1 protein expression in THP-1 with or without SW620 co-culture. [file Image_4.tif]
